# Interfacial interaction–driven rheological properties of quartz nanofluids from molecular dynamics simulations and density functional theory calculations

**DOI:** 10.1007/s00894-022-05177-w

**Published:** 2022-06-16

**Authors:** Zhaoyang Lou, Chen Cheng, Yingqi Cui, Hao Tian

**Affiliations:** 1 Department of Radiation Oncology, Affiliated Cancer Hospital of Zhengzhou University, Henan Cancer Hospital, Zhengzhou, China; 2 Institute of Atomic and Molecular Physics, Sichuan University, Chengdu, China; 3 Department of Strategy and Healthcare Development, Affiliated Cancer Hospital of Zhengzhou University, Henan Cancer Hospital, Zhengzhou, China

**Keywords:** Quartz nanofluids, Viscosity, Molecular dynamics simulation, Density functional theory calculation

## Abstract

Correlations of the shear viscosity of quartz nanofluids with particle concentration, particle size, and temperature were investigated with molecular dynamics simulations and density functional theory (DFT) calculations. A new understanding to the experimentally concluded correlations was addressed in terms of microscopic particle–water interfacial interaction in three aspects. First, the viscosity of quartz nanofluids at different particle concentrations, particle sizes, and temperatures were simulated using the equilibrium molecular dynamics simulations method to reproduce the experimental observations. At the same particle size, the nanofluid viscosity decreases significantly with temperature and increases with nanoparticle volume concentration, and at the same volume concentration, the nanofluid viscosity increases with the decrease of particle size. Second, DFT calculations confirm a stronger particle–water interaction than that among water molecules. The important role of particle–water interaction in the viscosity determination of nanofluids was revealed. Finally, a correlation was proposed to fit the simulated results and compared with earlier two-parameter correlations. One parameter in the correlation is indeed a constant, while the other is a function of SiO_2_–water interaction energy. Our study proposes a physical basis for the experimentally concluded correlations on the viscosity of nanofluids.

**Supplementary Information:**

The online version contains supplementary material available at 10.1007/s00894-022-05177-w.

## Introduction

Nanofluids are usually engineered by dispersing nano-sized particles in a base fluid such as water, ethylene glycol, propylene glycol, and so on. In the past decades, nanofluids were broadly used in many industries including power generation, chemical processes, heating and cooling processes, transportation, microelectronics, and other micro-sized applications because of their enhanced thermal and mechanical properties [1–5]. In fact, most of these applications make use of the unique rheological properties of nanofluids that vary with particle type, size, shape, and amount, in addition to the intrinsic properties of base fluids. Knowledge of the rheological behavior of nanofluids are therefore crucial in shaping their practical applications.

Many studies have been carried out on the rheological properties of nanofluids [6–14]. Minakov [6] systematically measured the viscosity of more than 30 different nanofluids based on distilled water, ethylene glycol, and engine oil, suggesting that their viscosity increases with decreasing particle diameter. Ezekwem [7] proposed a relationship of viscosity with temperature and volume concentration for AlN and SiC nanofluids. The nanofluid viscosity decreases significantly with temperature and increases with nanoparticle volume concentration. Pak [8] have studied experimentally γ-Al_2_O_3_-water and TiO_2_-water nanofluids and found their viscosity increases with increasing particle concentration. Moreover, the viscosity magnitudes are significantly greater than those from Batchelor equation [9]. Wang [10] reported a maximum enhancement of 86% for the viscosity of Al_2_O_3_ (28 nm)–water nanofluids. Similar viscosity increments were also observed by Wole-Osho [15]. In a study for CuO nanofluids, Kulkarni [12] correlated the viscosity, temperature and particle concentration in the form of $\mathrm{ln}{\mu }_{s}=A/T-B$, where *μ*_s_ is the suspension viscosity and *A* and *B* are two parameters related to volume concentration.

Even though these studies have revealed the rheological dependence of some nanofluids from macroscopic measurements, little is known about the molecular mechanism of how particle size and concentration affect the rheological properties of nanofluids. While the particle size and shape distributions are difficult to control in experiments, computer simulations provide a useful approach to explore such correlations explicitly. As molecular dynamics (MD) methods have been proven to be an effective and reliable approach to investigate the microscopic structures, rheological, and related properties of various gases, liquids, and solids [16–20], we carried out equilibrium molecular dynamics simulations and density functional theory (DFT) calculations on the quartz-in-water nanofluids in this work, aiming to reveal the origin of their viscosity dependence on particle size, particle concentration, and temperature. Quartz, abundant on the earth and easy in pulverization, is commonly used in the preparation of nano-additives. The addition of quartz nanoparticles leads to various improvements in rubber, plastic, and coating products. In most processes, quartz nanoparticles are dispersed into water or other fluids for ease of usage. Understanding to the rheological behavior of quartz nanofluids from their molecular mechanism could be helpful with their applications in manufacture and for the development of other kinds of nanofluids.

## Materials and simulation methods

Two approaches, equilibrium and non-equilibrium, were often used to calculate the viscosity in MD simulations [17, 20–23]. The non-equilibrium approach had ever been considered more efficient from a computational point of view than the equilibrium one that sometimes suffers from poorly converged viscosity. However, Chen [23] and Guo [24] have clarified that the convergence issues can be addressed with enough statistics and by a careful selection of the integration times. The equilibrium approach does not suffer from additional adjustments that the non-equilibrium approach usually needs. In addition, the equilibrium MD is a multi-property method with which all thermodynamic properties can be computed at the same state point from a single simulation run. Using an equilibrium simulation method, Wang [25] has studied the mechanism of heat flow in a model nanofluid system. The equilibrium MD simulations were therefore employed in this work. The viscosity is calculated by the Green–Kubo integral formula [26, 27]:

1 $${\mu }_{s}=\frac{V}{{k}_{B}T}{\int }_{0}^{\infty }\langle {P}_{\alpha \beta }(t){P}_{\alpha \beta }(0)\rangle dt$$

in the equilibrium approach. *μ*_*s*_ is the shear viscosity, *V* is the volume of the system, *T* is the temperature, *k*_*B*_ is the Boltzmann constant, and *P*_*αβ*_ are off-diagonal components of the pressure tensor.

**Fig. 1:**
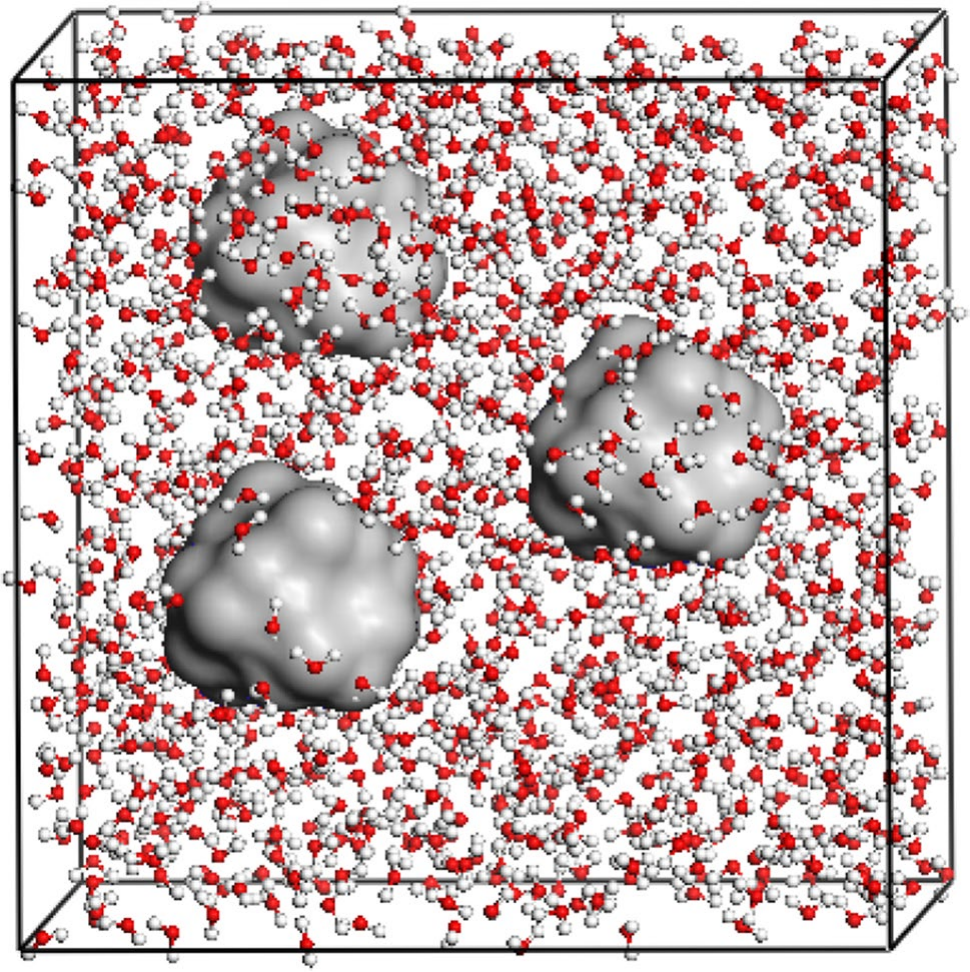
A snapshot of SiO_2_ particles (grey balls) in a cubic box filled with water

A periodic 30 × 30 × 30 Å^3^ cell was built to mimic the quartz–water system. The SiO_2_ particles were cut in the shape of a cube from bulk α-quartz. The Si and O atoms were arranged alternatively on the particle surface. The SiO_2_ particles were then randomly placed in the cell and the rest of the space was homogeneously filled with water molecules. The water density was set to 1.0 g/mL for all the starting structures. The diameter (the diagonal length of the cube) and number of SiO_2_ particles can be tuned to model the nanofluids with different concentrations. A snapshot of the SiO_2_–water cell is shown in Fig. 1. In the MD simulation, the TIP4P/2005 [28] force field for water and the CLAYFF [29] force field for SiO_2_ were used. The former has been widely used in the simulations for water-containing systems [30–32], and the latter was developed for clay systems and has shown great feasibility for water/clay systems [33–35]. A typical simulation was carried out in three successive steps with the LAMMPS package [36]. First, the system was subjected to an NPT dynamics for 800 ps using a time step of 1 fs at the temperature of interest. In this step, the cell volume was adjusted to match the density of the system at the target temperature. The system was then equilibrated for more than 100 ps in an NVT ensemble until its energy fluctuation becomes stable. Finally, the production steps of 10 ns were performed.

The simulations were conducted over a temperature range of 280–340 K, which was of interest in practical applications. A Nose–Hoover thermostat [37] was used to maintain the system temperature. Long-range electrostatic interactions were computed using the Particle–Particle Particle-Mesh K-space technique, and a cut-off of 10 Å was used for short-range interactions. The components of pressure tensor were accumulated at every time step. The pressure autocorrelation functions (ACFs) and the shear viscosity coefficient were then obtained by numerically integrating the components over a time window of 5 ~ 10 ps depending on the systems.

With these settings, we computed the shear viscosity of water, which is the solvent of quartz nanofluids, to validate the above computational strategy. The simulations were carried out over 280–340 K with an interval of 10 K. The results, as shown in Table 1, are in good agreement with previous experiments [38] for both shear viscosity and density. The maximum deviations are less than 5%.

**Table 1:** Calculated viscosities and density of the EMD simulations

	This work		Experimental [38]	
Temp	Viscosity	Density	Viscosity	Density
K	*μ*_*w*_/mPa·s	g/cm^3^	*μ*_*w*_/mPa·s	g/cm^3^
280	1.357 ± 0.032	0.986	1.428 (280.16 K)	1.000
290	1.031 ± 0.041	0.978	1.081 (290.16 K)	0.999
300	0.846 ± 0.016	0.980	0.851 (300.16 K)	0.997
310	0.667 ± 0.009	0.980	0.692 (310.16 K)	0.993
320	0.538 ± 0.029	0.980	0.576 (320.16 K)	0.989
330	0.486 ± 0.025	0.976	0.488 (330.16 K)	0.985
340	0.438 ± 0.023	0.965	0.421 (340.16 K)	0.979

The DFT calculations were carried out under the generalized gradient approximation (GGA) of Perdew–Burke–Ernzerhof (PBE) parameterization [39], as implemented in the DMol^3^ package [40]. Both the cluster model and the slab model geometry optimization calculations were performed using the double numerical polarization (DNP) basis set [40] which considers a polarization *d* function on heavy atoms and a polarization *p* function on hydrogen atoms. A Grimme-type [41] dispersion potential was used to describe the interatomic weak interaction in the systems. Periodic boundary condition was applied to the slab model to mimic the interactions between water molecule and surface. The slabs were separated from their images in the neighboring cells by a vacuum width of 30 Å, a distance large enough to avoid the interactions between neighboring slabs. For all the geometry optimizations, the convergence criteria were set to 0.004 a.u. on the gradient, 0.005 a.u. on the displacement, and 2.0 × 10^−5^ a.u. on the energy.

## Results and discussion

**Fig. 2:**
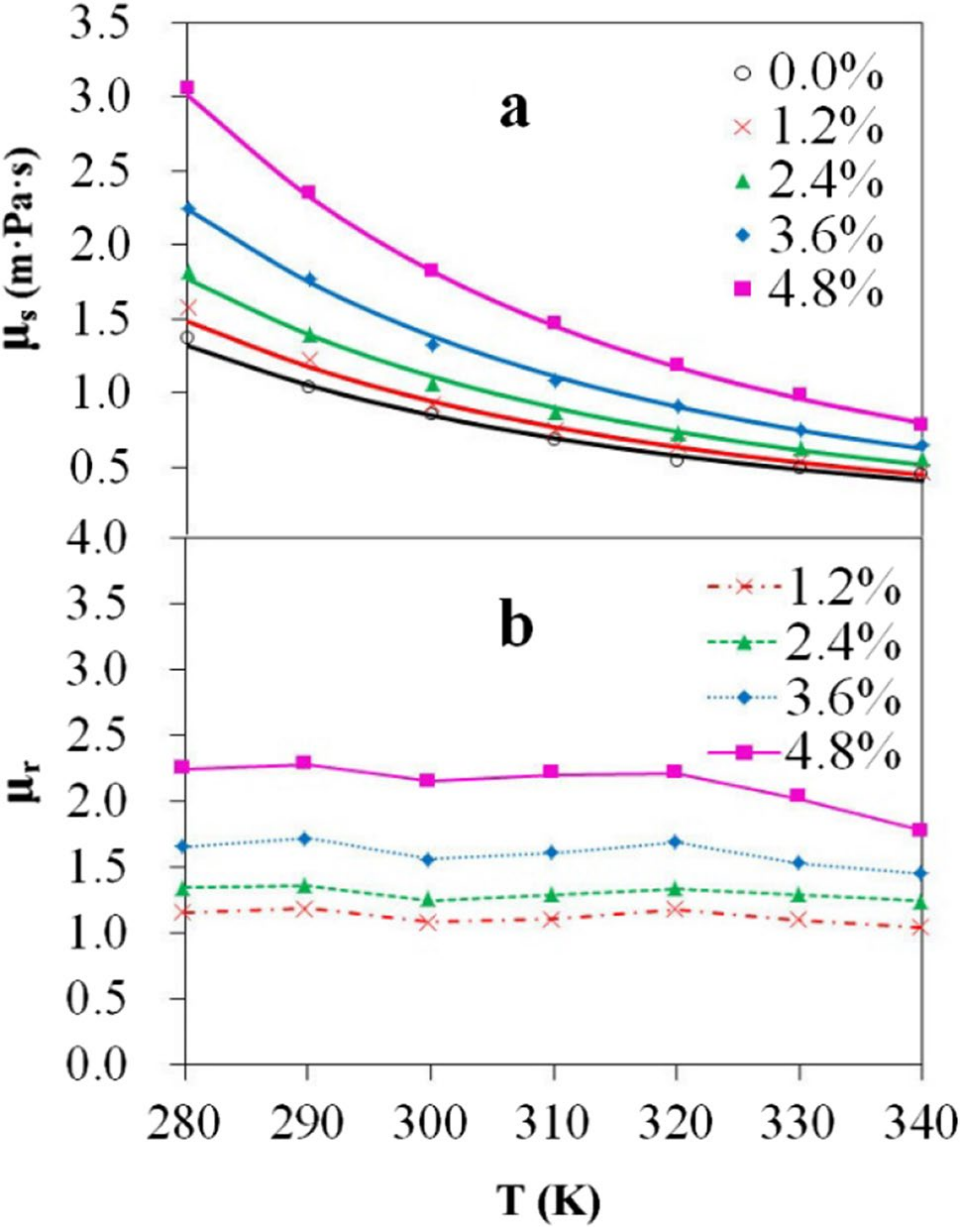
Computed viscosity (**a**) and relative viscosity (**b**) of quartz nanofluids at different volume concentrations and temperatures

The viscosity of quartz nanofluids was simulated at constant temperatures for the systems with different volume concentrations, which are defined as the volume fraction of SiO_2_ particles. As shown in Fig. 2a, the viscosity increases with SiO_2_ concentration at a given temperature (The detailed calculated viscosities of quartz nanofluids are shown in Supplementary Table S1). Moreover, the increase is more significant at lower temperatures. When the concentration increases from 1.2 to 4.8%, for example, the viscosity increases by 1.47 mPa·s at 280 K and 0.32 mPa·s at 340 K, respectively. For these simulations, all the SiO_2_ particles are fixed at 11.2 Å in diameter. A large volume concentration means more particles in the system. The increasing SiO_2_ particle number increases the particle–water contacting area, leading to increasing contribution from particle-water interaction to the viscosity. As we will show below, the particle–water interaction, which is stronger than water–water interaction, tends to increase the viscosity. The volume concentration dependence of nanofluids has been studied experimentally for quartz, copper oxide, and titanium dioxide systems [4, 13, 42, 43]. In Namburu’s measurements [42], the high concentrations of quartz nanoparticles in ethylene glycol and water mixture leads to great viscosity, and the viscosity variations at low temperature are more significant than that at high temperature. Our calculations produced similar results with the observations.

The temperature dependence of fluids has been well addressed by many authors [4, 11, 12, 42–44]. Increased molecular kinetic energy at high temperature usually makes the fluid viscosity small. This is true for quartz nanofluid. Figure 2 a also shows the temperature dependence of viscosity for the nanofluid at a given SiO_2_ volume concentration. Similar to water, the viscosity of quartz nanofluid decreases with temperature. However, the temperature sensitivity of viscosity is different for the systems. The viscosity of systems with higher SiO_2_ concentration drops more rapidly with temperature. In Fig. 2a, the slope of viscosity–temperature curves increases with SiO_2_ concentration, indicating that the SiO_2_–water interaction is more important in viscosity contribution at lower temperature. This holds only when the interaction between SiO_2_-water interfaces is stronger than that between water molecules.

**Fig. 3:**
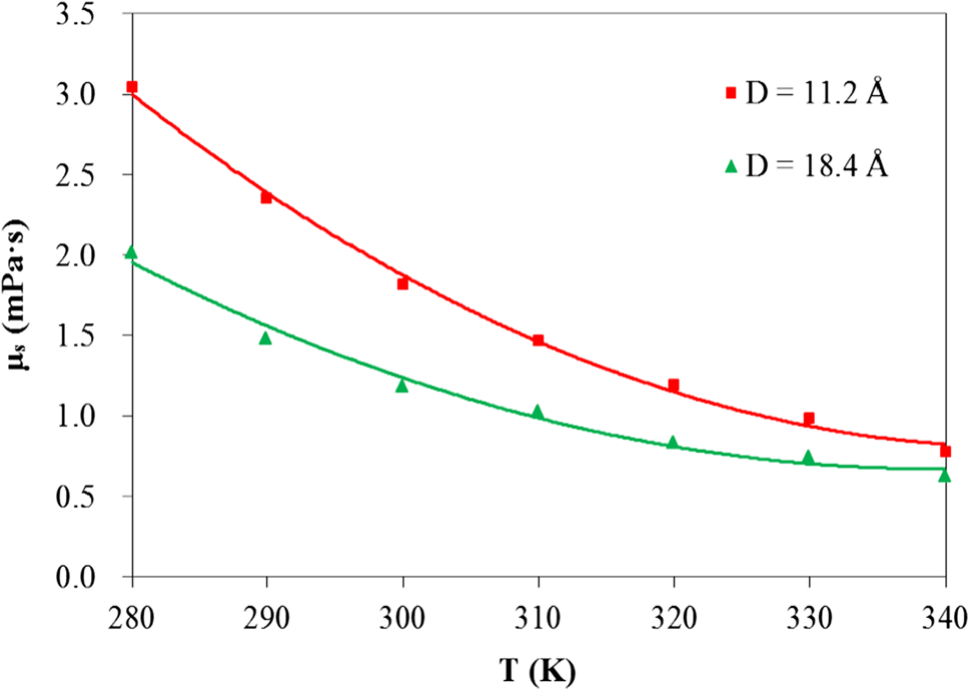
Computed viscosity of quartz nanofluids with different particle diameters

For quartz nanofluids with a given volume concentration, their viscosity depends on the particle size. In Fig. 3, we compare the viscosity of quartz nanofluids with different particle diameters. The large particle, about 18.4 Å in diameter, is larger than the small one (11.2 Å) by 170% in surface area and by 343% in volume. Since the total volume concentration of SiO_2_ particles is fixed, the system with smaller particle size has greater particle number. As a result, the total surface area increases by a ratio of 18.4:11.2. Therefore, SiO_2_–water interface interaction plays a more significant role in the system with smaller particle size. Our calculations reveal that at every temperature, the system with smaller particles has larger viscosity. Moreover, the difference becomes more remarkable at lower temperature. For example, the small SiO_2_ particles result in a viscosity of 0.78 mPa·s at 340 K, only 0.15 mPa·s larger than that by the large particles. At 280 K, however, their viscosity difference becomes 1.03 mPa·s. Namburu [42] measured the viscosity of SiO_2_ nanoparticles with various diameters of 20, 50, and 100 nm suspended in a 60:40 (by weight) ethylene glycol and water mixture in a wide temperature range from − 35 to 50 °C, revealing that at same volume concentration, the nanofluids with large particle diameters have low viscosity. Our calculated results are consistent with the experimental observations for both the particle size dependence and its variation with temperature.

It is interesting to look into the relative viscosity, which was often used to measure the viscosity of nanofluids. The relative viscosity is defined as the viscosity ratio of nanofluid with respect to pure solvent, *μ*_*r*_ = *μ*_*s*_*/μ*_*w*_, where *μ*_*w*_ is the viscosity of water. The computed *μ*_*r*_ values for quartz nanofluids are shown in Fig. 2b. Remarkable concentration dependence can be noted. The ratios are about 1.1, 1.3, 1.6, and 2.2 for the four concentrations, and nearly unchanged within the temperature range except that for the highest concentration of 4.8%. Such terraced increase of *μ*_*r*_ with respect to concentration confirms that the viscosity increase mainly comes from SiO_2_–water interaction rather than water–water or SiO_2_–SiO_2_ interaction. It has been observed that the relative viscosity of copper oxide nanofluid has very small changes (less than 0.3) over − 35 to 50 °C at low concentrations [43]. The *μ*_*r*_ decay at high temperature for the samples with high concentrations was also noted. Similar results were also reported by Prasher for alumina particles suspended in propylene glycol with a volume concentration of 0.5%, 2%, and 3% at 30–50 °C [11]. Our calculations reveal that the *μ*_*r*_ of quartz nanofluids are concentration dependent instead of temperature dependent at low SiO_2_ concentrations. In addition, we also noted the decay of *μ*_*r*_ at high temperature (above 300 K) for systems with relatively high volume concentrations of 3.6% and 4.8%.

**Fig. 4:**
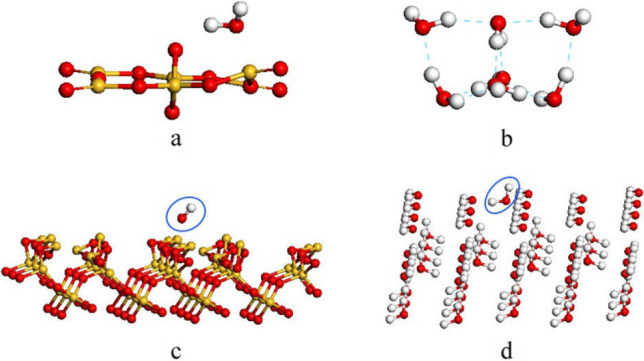
Structure models for DFT calculations. A water molecule adsorption on a (SiO_2_)_6_ cluster (**a**), (H_2_O)_6_ cluster (**b**), SiO_2_ (001) surface (**c**), and ice (001) surface (**d**)

The above calculations demonstrate the important role of SiO_2_-water interaction. It is therefore interesting to inspect such kind of interaction further. Two computational models were then designed to evaluate the interacting patterns between SiO_2_ and water by means of DFT calculations, as shown in Fig. 4. One is the cluster model in which a water molecule adsorbs onto a (SiO_2_)_6_ cluster. The structure of (SiO_2_)_6_ cluster was taken from Ref. [45]. The water H atom binds with one of the O atoms of the cluster via a hydrogen bond and with a bond distance of 1.77 Å. The interaction energy, which is defined as the energy difference between the systems before and after water adsorption, is about 1.43 eV. A similar cluster model, a water molecule adsorbing onto a (H_2_O)_6_ cluster, which was taken from Ref. [46], gives the interaction energy of 0.65 eV between the water molecule and the water cluster. In the second model, periodical DFT calculations were performed to compute the interaction of a water molecule on the SiO_2_ (001) and ice (001) surfaces, which were sliced respectively from α-quartz and cube-ice crystal structures. Under this slab model, the computed interaction energy is 1.78 eV for a water molecule on the SiO_2_ surface and 0.94 eV on the ice surface. Larger interaction energies between SiO_2_ and water were predicted by both the cluster and the slab models, confirming above speculations from MD computations at the force-field level.

**Fig. 5:**
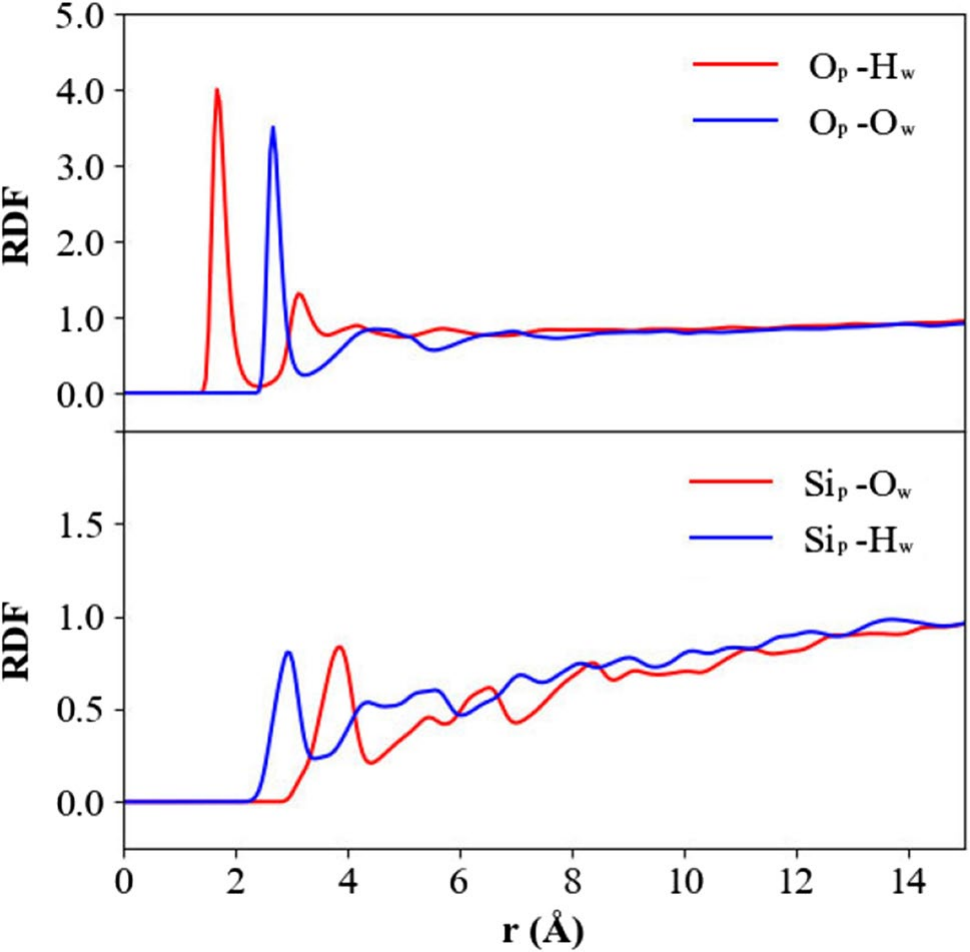
Radial distribution functions between surface atoms of quartz nanoparticles and atoms of water molecule at 300 K, O_p_ represents the oxygen atom of nanoparticle, Si_p_ represents the silicon atom of nanoparticle, O_w_ represents the oxygen atom of water molecule, H_w_ represents the hydrogen atom of water molecule

The radial distribution function (RDF) was computed to analyze the affinity between surface atoms of quartz nanoparticles and atoms of water molecule. As is shown in Fig. 5, the RDF between H_w_ (hydrogen atom of water molecule) and O_p_ (oxygen atom of nanoparticle) atoms in the first peak is sharper than the values of other pairs, indicating that water molecule is preferentially adsorbed on the sites of oxygen atoms of nanoparticle. Meanwhile, we observe that the first peak is at *r* ≈ 1.69 Å; this distance is in accordance with the hydrogen bond distance of water molecule adsorbed on (SiO_2_)_6_ cluster. The first peaks of RDFs between O_p_ and O_w_ atoms are higher than the Si_p_–O_w_ and Si_p_–H_w_ first peaks, which also suggests that water molecule preferentially occupies the sites of oxygen atoms of nanoparticle.

The phenomenon that the viscosity of quartz nanofluids increases can be explained as follows: from the microscopic point of view, a network structure connected by intermolecular interaction is formed in the solution. Due to the strong interaction between water molecules and quartz nanoparticles, the internal friction of nanofluids increases, resulting in the increase of viscosity. When the size of nanoparticles is the same, the increase of volume concentration leads to the increase of the interaction between water molecules and nanoparticles per unit volume, which is manifested as the increase of viscosity. When the volume concentration is the same, with the decrease of particle size, the number and surface area of nanoparticles per unit volume increase significantly, which also leads to the increase of the interaction between water molecules and nanoparticles, and then leads to the increase of the viscosity of nanofluids.

Several expressions have been proposed by Bicerano [47], Brinkman [48], Duangthongsuk [4], Kulkarni [12], and Namburu [43] to fit the measured viscosity data of nanofluids, providing an estimation for viscosity variation with particle concentration and/or temperature. Most of these correlations are similar in nature, though different parameters were used to adjust the values for high-concentration systems. The effect of particle size, however, is ignored in these correlations. As we found above, the nanofluid systems with different particle sizes may have quite different viscosities even though they have the same volume concentration. Our MD and DFT calculations revealed the decisive role of SiO_2_-water interaction in the rheological behavior of quartz nanofluids. We would explore below the correlation of the viscosity of quartz nanofluids with particle-water interaction strength.

**Fig. 6:**
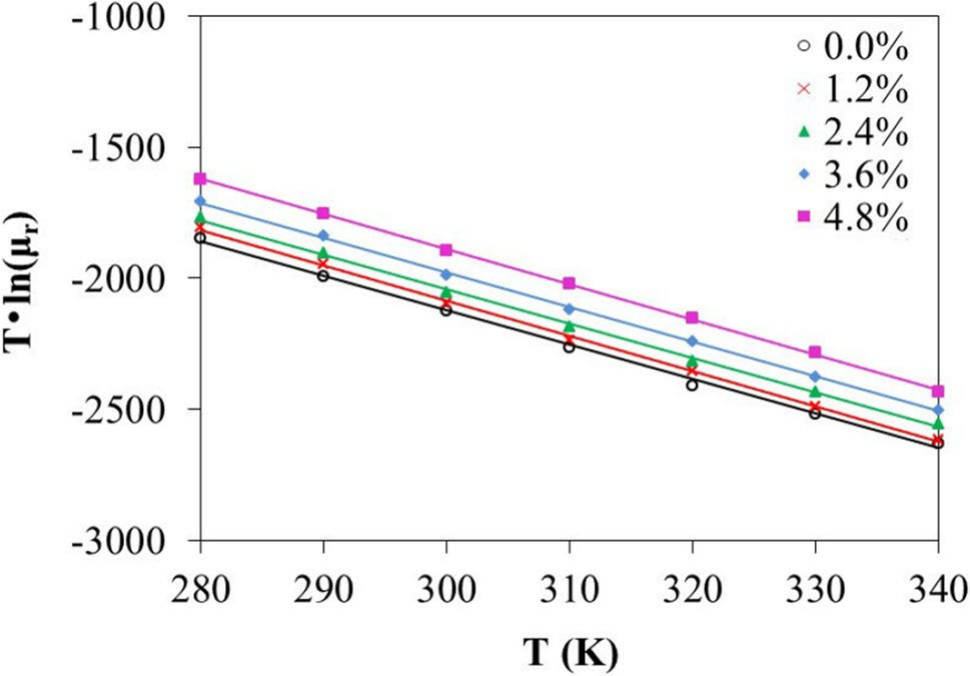
Correlation of T·ln(*μ*_*r*_) with temperature (*T*) fitted with formula (3)a

Starting from the data in Fig. 2, an exponential correlation,

2 $${\mu }_{s}=A{e}^{-B/T}$$

can be fitted. Formula (2) is the so-called Arrhenius equation [49]. For nanofluids, *A* and *B* are the polynomials about volume concentration of nanofluids. In this work, the fitting of *A* and *B* was achieved through numpy polynomial module [50]; a good correlation with *R*^2^ > 0.99 is obtained, as shown in Fig. 6 and Supplementary Table S2 and Supplementary Fig S1. Both *A* and *B* are 2nd-order polynomials, as is shown in formulas (2)a and (2)b:

2a $$A=0.5735{\phi }^{2}-0.002915\phi +0.00106$$

2b $$B=-3.649{\phi }^{2}-77.88\phi -2009$$

where $\phi$ represents the volume concentration. From formula (2), one has

2c $${\mathrm{ln}\mu }_{s}=\mathrm{ln}A-{~}^{B}\!\left/ \!{~}_{T}\right.$$

**Fig. 7:**
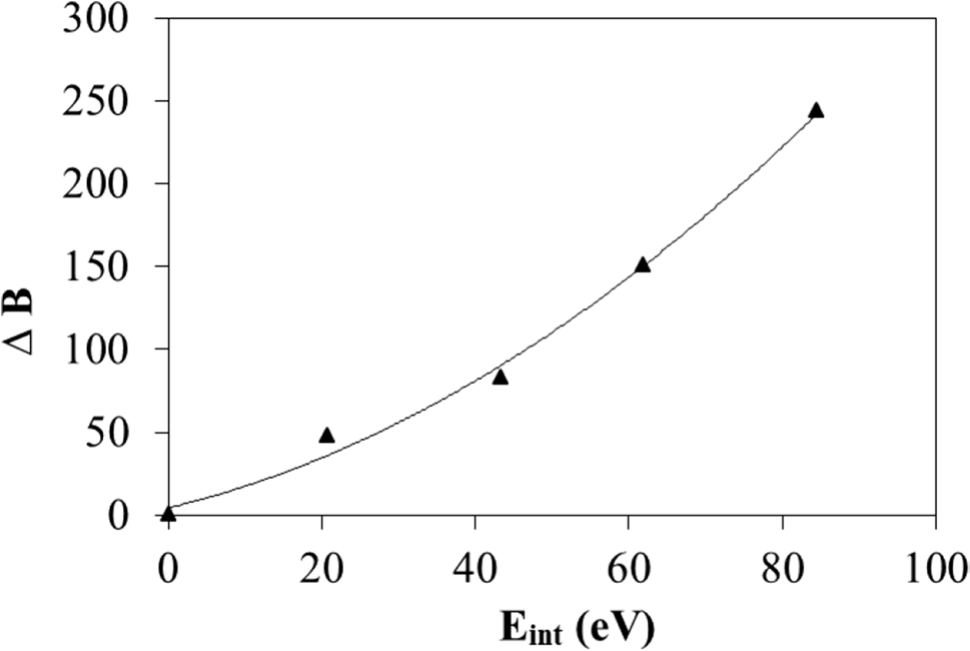
Correlation of Δ*B* with SiO_2_–water interaction energy (*E*_*int*_)

Thus,

3 $$\mathrm{ln}{\mu }_{r}={A}^{^{\prime}}-\Delta B/T$$

3a $$T\bullet \mathrm{ln}{\mu }_{r}={T\bullet A}^{^{\prime}}-\Delta B$$

where *A´* = ln *A* − ln *A*_*w*_, and Δ*B* = *B* − *B*_*w*_. *A*_*w*_ and *B*_*w*_ is fitted from the viscosity of pure water. Figure 6 is drawn from formula (3)a. Figure 6 shows that the systems with different SiO_2_ volume concentrations have similar slopes (*A′*). The concentration effect on the viscosity is then represented only in parameter *B*. Formula (3) has the similar form with the correlations proposed by Kulkarni [12] and Namburu [43] in which both *A* and *B* were functions of particle concentrations. From our MD simulations, the SiO_2_–water interaction energy (*E*_*int*_) can be obtained by summing up the coulomb and van der Waals terms between SiO_2_ particles and water molecules. Figure 7 presents the correlation of Δ*B* with *E*_*int*_. It is interesting to note that with increasing SiO_2_-water interaction energy Δ*B* increases, leading to increasing *μ*_*r*_. Therefore, the concentration-dependent parameters in previously observed correlations [12, 43] for nanofluids can be further understood as quantities relating to particle-solute interaction and can be expressed as functions of interaction energy.

## Conclusion

The rheological properties, in particular to the viscosity variations, of quartz nanofluids with particle concentration, particle size, and temperature were simulated using the equilibrium molecular dynamics method and density functional theory calculations. Our calculations reproduce the experimental observations that the viscosity of quartz nanofluids increases with particle concentration and decreases with temperature. At a fixed volume concentration, moreover, the viscosity increases with decreasing particle size. The viscosity variations were rationalized in terms of the particle–water and water–water interactions in the nanofluid systems. DFT calculations with both cluster and slab models reveal that the interaction between SiO_2_ particle and water is much stronger than that between water molecules, which is responsible for the viscosity variation of quartz nanofluids. Increasing the volume concentration of particle with the same size and reducing the size of particle with the same volume concentration of nanofluids will increase the interaction between water molecules and SiO_2_ particles, resulting in the increase of the viscosity of nanofluids. Furthermore, a correlation was proposed to fit the simulated results and compared with earlier correlations. A new understanding to the parameters in previously observed correlations was proposed from microscopic particle–water interfacial interaction. One parameter is a constant, while the other is a function of SiO_2_–water interaction energy.

## Supplementary Information

Below is the link to the electronic supplementary material.

Supplementary file1 (DOCX 143 KB)

## Data Availability

All data generated or analyzed during this study are included in this published article.
