# Supplementary material for: Interfacial interaction–driven rheological properties of quartz nanofluids from molecular dynamics simulations and density functional theory calculations
Source: J Mol Model. 2022 Jun 16;28(7):189. doi: 10.1007/s00894-022-05177-w (PMC9203409; doi:10.1007/s00894-022-05177-w)
Supplement: Supplementary file 1 — Supplementary file1 (DOCX 143 KB) [file 894_2022_5177_MOESM1_ESM.docx]

**Interfacial interaction driven rheological properties of** **quartz nanofluids from molecular dynamics simulations and density functional theory calculations**

Zhaoyang Lou^a,c*^, Chen Cheng^b^, Yingqi Cui^c^, Hao Tian^c^

^a^ Department of Radiation Oncology, Affiliated Cancer Hospital of Zhengzhou University, Henan Cancer Hospital, Zhengzhou, China; ^b^ Department of Strategy and Healthcare Development, Affiliated Cancer Hospital of Zhengzhou University, Henan Cancer Hospital, Zhengzhou, China; ^c^ Institute of Atomic and Molecular Physics, Sichuan University, Chengdu, China

***corresponding author:**

Zhaoyang Lou

louslove@163.com

**Supporting Information**

**Table S1** Simulated viscosities of quartz nanofluids with different volume concentration and temperature.

| Temp/K | Viscosity/mPa·s (D=11.2 Å) | | | | | Viscosity/mPa·s (D=18.4 Å) |
| --- | --- | --- | --- | --- | --- | --- |
|  | 0.00% | 1.20% | 2.40% | 3.60% | 4.80% | 4.80% |
| 280 | 1.357±0.032 | 1.557±0.043 | 1.764±0.048 | 2.226±0.051 | 3.051±0.053 | 2.022±0.057 |
| 290 | 1.031±0.041 | 1.198±0.027 | 1.365±0.044 | 1.748±0.050 | 2.357±0.048 | 1.478±0.043 |
| 300 | 0.846±0.016 | 0.895±0.036 | 1.023±0.037 | 1.302±0.044 | 1.819±0.045 | 1.181±0.046 |
| 310 | 0.667±0.009 | 0.720±0.022 | 0.831±0.031 | 1.062±0.033 | 1.472±0.035 | 1.023±0.037 |
| 320 | 0.538±0.029 | 0.608±0.032 | 0.688±0.026 | 0.887±0.028 | 1.194±0.033 | 0.828±0.031 |
| 330 | 0.486±0.025 | 0.505±0.029 | 0.576±0.032 | 0.712±0.024 | 0.979±0.024 | 0.752±0.028 |
| 340 | 0.438±0.023 | 0.417±0.025 | 0.489±0.024 | 0.616±0.016 | 0.771±0.022 | 0.625±0.027 |

**Table S2** Calculated viscosities of quartz nanofluids through formular (2).

| Temp/K | Viscosity/mPa·s (D=11.2 Å) | | | | |
| --- | --- | --- | --- | --- | --- |
|  | 0.00% | 1.20% | 2.40% | 3.60% | 4.80% |
| 280 | 1.385 | 1.452 | 1.736 | 2.241 | 2.968 |
| 290 | 1.081 | 1.133 | 1.356 | 1.749 | 2.316 |
| 300 | 0.858 | 0.900 | 1.076 | 1.388 | 1.838 |
| 310 | 0.692 | 0.725 | 0.867 | 1.118 | 1.480 |
| 320 | 0.565 | 0.592 | 0.708 | 0.913 | 1.208 |
| 330 | 0.467 | 0.489 | 0.585 | 0.754 | 0.999 |
| 340 | 0.390 | 0.409 | 0.489 | 0.631 | 0.835 |


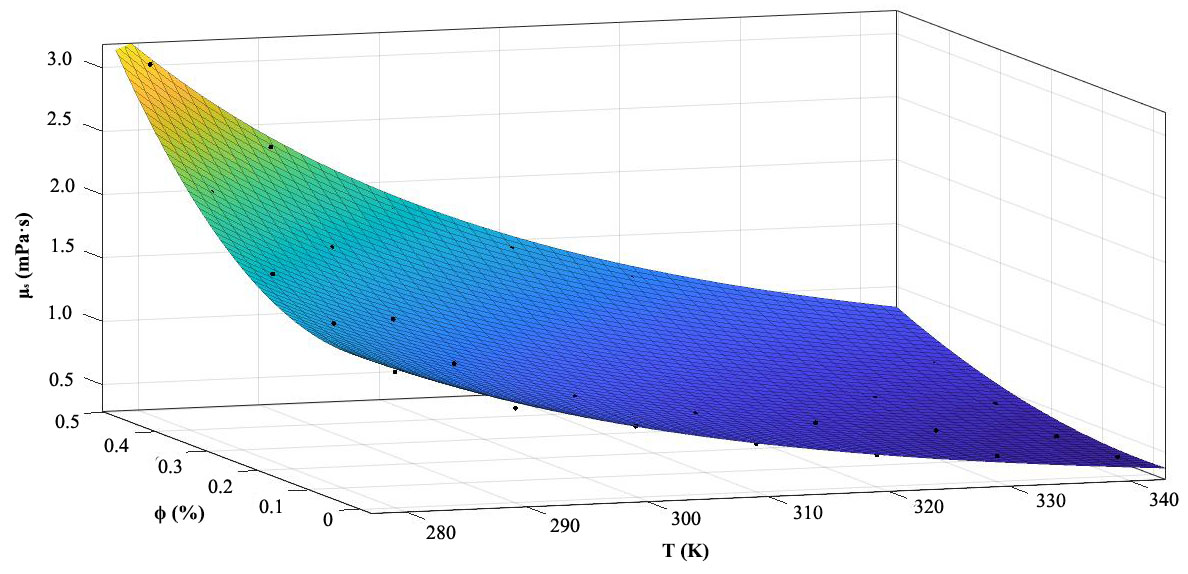


**Fig S1** Comparison of viscosities metrics between calculated values through formular 2 (colored surface) and simulated values (black dots) through molecular dynamics modeling, with R^2^ = 0.996.
